# Comparison of Primary Models to Predict Microbial Growth by the Plate Count and Absorbance Methods

**DOI:** 10.1155/2015/365025

**Published:** 2015-10-11

**Authors:** María-Leonor Pla, Sandra Oltra, María-Dolores Esteban, Santiago Andreu, Alfredo Palop

**Affiliations:** ^1^Departamento de Matemática Aplicada, EPSA, Universidad Politécnica de Valencia, Plaza Ferrandiz y Carbonell, s/n, 03801 Alcoy, Spain; ^2^Departamento de Ingeniería de Alimentos y del Equipamiento Agrícola, Universidad Politécnica de Cartagena, Campus de Excelencia Internacional Regional “Campus Mare Nostrum”, Paseo Alfonso XIII 48, 30203 Cartagena, Spain; ^3^Instituto de Biotecnología Vegetal, Universidad Politécnica de Cartagena, Campus de Excelencia Internacional Regional “Campus Mare Nostrum”, Edificio I+D+I, Muralla del Mar, 30202 Cartagena, Spain

## Abstract

The selection of a primary model to describe microbial growth in predictive food microbiology often appears to be subjective. The objective of this research was to check the performance of different mathematical models in predicting growth parameters, both by absorbance and plate count methods. For this purpose, growth curves of three different microorganisms (*Bacillus cereus, Listeria monocytogenes*, and *Escherichia coli*) grown under the same conditions, but with different initial concentrations each, were analysed. When measuring the microbial growth of each microorganism by optical density, almost all models provided quite high goodness of fit (*r* ^2^ > 0.93) for all growth curves. The growth rate remained approximately constant for all growth curves of each microorganism, when considering one growth model, but differences were found among models. Three-phase linear model provided the lowest variation for growth rate values for all three microorganisms. Baranyi model gave a variation marginally higher, despite a much better overall fitting. When measuring the microbial growth by plate count, similar results were obtained. These results provide insight into predictive microbiology and will help food microbiologists and researchers to choose the proper primary growth predictive model.

## 1. Introduction

Predictive microbiology enables, through the use of mathematical models, estimating the behaviour of microorganisms under certain circumstances [1], based upon the premise that the responses of microorganisms to environmental factors are reproducible. The ability to predict both the growth of microorganisms, as affected by different environmental factors, and the survival of microorganisms as a result of preservative treatments is an important tool for evaluating the safety and shelf life of food products.

Before predictive microbiology can be applied to the food industry, mathematical models that adequately describe microbial behaviour are needed. There are a number of sigmoid equations and several models that have been used as growth functions. They all differ in “ease of use” and number of parameters in the equation. Some authors have compared the behaviour of different growth models, from different viewpoints, including mathematical measures of goodness of fit [2] and/or other statistical criteria [3–5]. The usual measures of goodness of fit for model comparison in previous studies were done by calculating the bias (*B*_*f*_) and accuracy (*A*_*f*_) indices as provided by Ross [6], the coefficient of determination (*r* ^2^), the residual mean square error (RMSE), or the *F*-test. Other authors [7, 8] have focussed on direct comparisons of particular growth parameters as predicted by various models.

These studies have reached different conclusions. Hence, there is significant disagreement in literature on which is the best-fitting model for predictive microbiology. The selection of a model in predictive food microbiology often appears to be subjective. Based on reports in the literature, Gompertz, Baranyi, Richards, logistic, and three-phase linear models are the most widely used [5, 9–11].

The growth curve has been mostly expressed in terms of microbial numbers (concentration of colony forming units), but also in terms of optical density as an indirect measurement. The measurement of absorbance is a rapid, nondestructive, inexpensive, and relatively easy-to-automate method to monitor bacterial growth, as compared to many other techniques and particularly when compared to classical viable count methods. When modelling optical density growth curves, the fitted parameters are different from the population growth parameters derived from viable counts. The rate of increase of the optical density does not express the maximum specific growth rate and the detection time is not equivalent to the lag time, unless the initial inoculum is greater than the detection limit. In spite of the limitations related to detection thresholds, correlation with the parameters derived from viable count growth curves, and inability to model growth in turbid liquid foods and in solid food matrixes, numerous techniques and mathematical growth models have been used in recent years for estimation of growth rates and lag times from absorbance data [5, 7, 8, 12–14]. In the opinion of Dalgaard and Koutsoumanis [7], absorbance techniques should be limited to conditions where high cell densities are reached, such as those resembling the growth of spoilage bacteria in foods. Even assuming the limitations of absorbance to build growth curves, it may be useful, if not to obtain very precise growth kinetic parameters, at least to compare the growth of different cultures or of the same cultures but in different conditions.

Work modelling the behaviour of bacteria in foods has shown that the lag phase is more difficult to predict than is the specific growth rate [15], mainly because of the influence in lag time of the physiological state of individual bacterial cells and, to a minor extent, of the inoculum size. The physiological state of the cells is affected by their previous growth environment and by exposure to stress conditions, which can extend the lag time considerably and also increase individual cell lag time variability [16–18]. However, microorganisms with a similar precultural history exposed to the same favourable growth conditions should be in a similar optimum physiological state and, thus, its effect on lag time variability is negligible [19]. Regarding the inoculum size, Baranyi and Pin [20] showed that as the cell number in the inoculum decreases, the population lag increases by an amount that depends on the distribution of individual lag times and the maximum specific growth rate. Augustin et al. [21] showed that the inoculum level effect can be explained by an increasing variability in individual cell lag time when stress factors become more stringent, and Baranyi and Pin [20] found that, under optimum growth conditions, this effect would only be expected at inoculum levels below about 10^2^-10^3^ cells, because the impact of variability among a small population of cells can become more important on lag time [2, 22].

Hence, a sufficiently large population of microorganisms exposed to exactly the same favourable growth conditions and with a similar precultural history behave in a similar way; that is, they should show the same growth parameters, growth rate, and lag phase duration.

The objective of this research was to check the performance of different mathematical models in predicting growth parameters, by both absorbance and plate count methods. For this purpose, growth curves of three different microorganisms (*Bacillus cereus, Listeria monocytogenes, *and* Escherichia coli*) grown, each species, under the same conditions, but with different initial concentrations, were analysed.

## 2. Materials and Methods

### 2.1. Microorganisms

*Bacillus cereus, Listeria monocytogenes, *and* Escherichia coli* were chosen as representative microorganisms for spore-forming, Gram-positive, and Gram-negative bacteria, respectively.

*B. cereus* INRA-AVTZ415 was kindly provided by the Institut National de la Recherche Agronomique (INRA, Avignon, France).* L. monocytogenes *and* E. coli *type strains (CECT 4031 and CECT 515, resp.) were provided by the Spanish Type Culture Collection (CECT).

To inoculate the growth media,* B. cereus* vegetative cells were grown at 30°C in brain heart infusion broth (BHI; Scharlau Chemie S.A., Barcelona, Spain), until the stationary phase of growth was reached.* L. monocytogenes *vegetative cells were grown at 37°C in tryptic soy broth (TSB; Scharlau Chemie) supplemented (w/v) with 0.6% yeast extract (YE; Scharlau Chemie), until the stationary phase of growth was reached.* E. coli* vegetative cells were grown at 37°C in TSB+YE acidified to pH 5 with ClH (Panreac Química, Barcelona, Spain) until the stationary phase of growth was reached. These growth conditions were chosen as favorable for these microorganisms.

### 2.2. Optical Density Growth Curves

100-well microtitre plates were filled with 400 *μ*L of the growth media (BHI for* B. cereus, *TSB+YE for* L. monocytogenes, *and pH 5 TSB+YE for* E. coli*) and were inoculated with the microorganisms and incubated in a Bioscreen C analyzer (Oy Growth Curves Ab Ltd., Helsinki, Finland). Initial concentrations in the growth media were 10^1^, 10^2^, 10^3^, 10^4^, 10^5^, and 10^6^ CFU mL^−1^ for* B. cereus, *10^2^, 10^3^, 10^4^, 10^5^, 10^6^, and 10^7^ CFU mL^−1^ for* L. monocytogenes,* and 10^2^, 10^4^, and 10^6^ CFU mL^−1^ for* E. coli*. In order to avoid variability derived from differences in the physiological state of different cultures, all the growth curves from each bacterium were obtained from a single bacterial culture. Growth media were incubated at 30°C for* B. cereus *and at 37°C for* L. monocytogenes *and* E. coli*. At 20 min intervals, the optical density (OD) of the samples using a wideband filter (420–580 nm) was measured.

For each combination of microorganism and initial concentration, 25–30 repetitions were performed, except for* B. cereus* 10^1^ and 10^2^ CFU mL^−1^ initial concentrations, where 13 and 21 repetitions were performed, and for* L. monocytogenes* 10^3^, 10^5^, and 10^7^ CFU mL^−1^ initial concentrations, where 5 repetitions each were performed. Growth curves were obtained by plotting the OD against the exposure time. A total of 345 individual growth curves were generated by absorbance measurements.

### 2.3. Plate Count Growth Curves

50 mL flasks of the growth media were inoculated with the microorganisms and incubated with agitation at 500 rpm. Growth media and incubation temperatures were the same as those used for optical density growth curves. Initial concentrations in the growth media were 10^1^, 10^3^, and 10^5^ CFU mL^−1^ for* B. cereus *and 10^2^, 10^4^, and 10^6^ CFU mL^−1^ for* L. monocytogenes *and* E. coli*. At preset time intervals, samples were taken, properly diluted in buffered peptone water (BPW, Scharlau Chemie), and incubated in BHI agar (BHIA, Scharlau Chemie) for 24 h at 30°C for* B. cereus *and in tryptic soy agar (TSA, Scharlau Chemie) + YE for 24 h at 37°C for* L. monocytogenes *and* E. coli*. Growth curves were performed in duplicate.

Growth curves were obtained by plotting log CFU mL^−1^ against the exposure time.

### 2.4. Mathematical Models

Analyses of the growth curves were performed using five primary growth models. These growth models were based on either linear (derived from the Monod model) or nonlinear (Gompertz, Logistic, Richards, and Baranyi) equations (Table 1) and reparameterized to reflect microbial growth parameters as derived by Zwietering et al. [3].

Curve fitting of three-phase linear, Gompertz, logistic, and Richards models was done using the curve-fitting tool of Matlab 7.0 (Math Works, Natick, USA) with which 95% confidence limits (CL) for growth parameters and *r* ^2^, RMSE, and sum of square error (SSE) of fit were calculated.

The curve fitting of Baranyi's equation was done using DMFit 2.0 program and the model of Baranyi and Roberts [23] as kindly provided by Dr. József Baranyi. This program provided standard error for each growth parameter and *r* ^2^ and standard error of fit. With these values, 95% CL of growth parameters and RMSE of fit were calculated.

Analysis of variance, medians, and quartiles for box and whisker plots were calculated using StatGraphics (StatPoint Technologies, Warrenton, USA). *p* values were always lower than 0.05.

## 3. Results and Discussion

### 3.1. Optical Density Growth Curves

**Figure 1:**
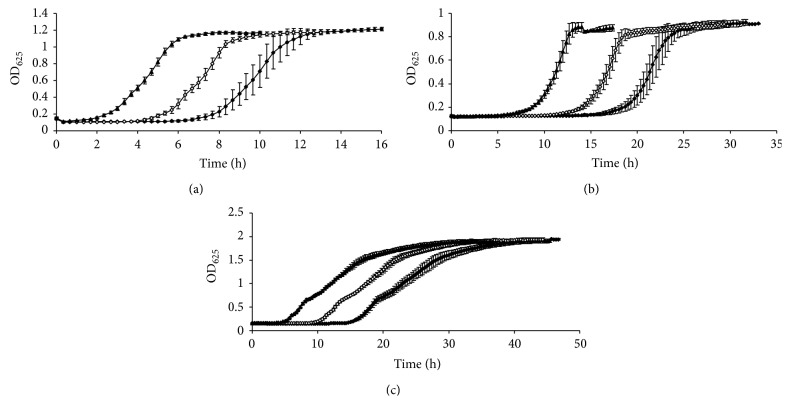
Optical density growth curves plotted with the average OD values (±standard deviation) at each sampling time of* Bacillus cereus *INRA-AVTZ 415 at 30°C in BHI (a),* Listeria monocytogenes *CECT 4031 at 37°C in TSB+YE (b), and* Escherichia coli *CECT 515 at 37°C in pH 5 TSB+YE (c). Initial number of microorganisms: (⧫) 10^2^ CFU mL^−1^; (◊) 10^4^ CFU mL^−1^; (▲) 10^6^ CFU mL^−1^.

Figure 1 shows the optical density growth curves plotted with the average OD values at each sampling time of* B. cereus *INRA-AVTZ 415 at 30°C in BHI (a),* L. monocytogenes *CECT 4031 at 37°C in TSB+YE (b), and* E. coli *CECT 515 at 37°C in pH 5 TSB+YE (c) starting at 10^2^, 10^4^, and 10^6^ CFU mL^−1^. These average growth curves correspond to 25–30 individual growth curves each. The slopes of all the growth curves corresponding to a microorganism were parallel in the exponential growth phase; that is, the growth rates were similar, as they should correspond to different cultures of the same microorganism growing exactly in the same conditions. However, a progressive decrease of lag phase duration was observed as the initial concentration increased. This decrease in lag time can be easily explained because the culture spends less time in reaching a concentration where the absorbance increases. Actually, several authors have recently used simple equations to derive real lag time and provided the initial concentration of microorganisms and the observed lag time from OD measurements [24–26]. The relatively low standard deviation values obtained, especially for growth curves starting at 10^4^ and 10^6^, are indicative of the repetitivity of the growth curves. It can be noticed that error bars are bigger as the initial concentration decreases, as a consequence of the increased variability among a small population of cells [2].

Table 2 shows the growth parameters given by three-phase linear, Gompertz, logistic, Richards, and Baranyi models for the growth curves of* B. cereus* plotted with the average OD values shown in Figure 1(a). All the models tested provided the values that could be expected for growth parameters of the three growth curves selected of this microorganism. Figure 1(a) shows that all three average growth curves of* B. cereus* were approximately parallel in their exponential growth phases. Although the curve starting at 10^2^ CFU mL^−1^ had a slightly lower slope, only logistic and Richards models showed significant differences between this and the other two curves of* B. cereus* starting at higher initial concentrations (Table 2). When comparing the growth rate values given by the different models, three-phase linear model gave the lowest values (0.27–0.30 OD units h^−1^), followed by Baranyi model (0.28–0.31 OD units h^−1^), logistic and Gompertz (0.31–0.35 OD units h^−1^), and Richards model (0.30–0.42 OD units h^−1^). Also, all models provided shorter lag phases at higher initial concentrations, as shown in Figure 1(a). Initial absorbance (*y*_0_) had values between 0.071 and 0.180 for all growth curves and models. The increase in absorbance from initial to final optical density (*C*) had values between 0.975 and 1.107 for all growth curves and models. Hence all models seemed to perform adequately, providing expected growth parameter values for these growth curves. *r* ^2^ values were higher than 0.995 for all growth curves and all models, except for three-phase linear model, which gave values as low as 0.989, hence being the model which provided worst fit to the data, as also it could be expected from a model consisting of three straight lines. All models gave RMSE values lower than 0.035.

Table 3 shows the growth parameters given by the five models for the growth curves of* L. monocytogenes* shown in Figure 1(b). Similar results were obtained, although in this case the only model which did not provide significant differences for the three growth rate values for the three initial concentrations tested was the three-phase linear. Again, the same order was obtained, three-phase linear model giving the lowest values (0.15–0.17 OD units h^−1^), followed by Baranyi model (0.15–0.20 OD units h^−1^), logistic and Gompertz (0.17–0.22 OD units h^−1^), and Richards model giving the highest values (0.18–0.32 OD units h^−1^). Also, all models provided shorter lag phases at higher initial concentrations. *y*_0_ values were between 0.119 and 0.149 and *C* values between 0.712 and 0.784. Again, *r* ^2^ values were higher than 0.990 for all growth curves and all models, except for three-phase linear model, which gave values as low as 0.930. Three-phase linear model also gave RMSE values as high as 0.061.

Table 4 shows the growth parameters given by the five models under study for the growth curves of* E. coli* shown in Figure 1(c). In this case, some unexpected results were obtained. Three-phase linear, Gompertz, logistic, and Baranyi models behaved as previously described for* B. cereus *and* L. monocytogenes*. Each model provided very similar values for the growth rates of the three growth curves, with only slight differences among them. However, only logistic and Baranyi models did not provide significant differences. For this microorganism, again, the three-phase linear model gave the lowest value for the growth rate (0.10–0.11 OD units h^−1^), but in this case together with Baranyi model, and again logistic and Gompertz models gave higher values (0.12–0.13 OD units h^−1^). However, Richards model gave unexpectedly low values for growth rates (<0.001 OD units h^−1^). All models provided shorter lag phases at higher initial concentrations. *y*_0_ values were between 0.060 and 0.176 for all growth curves and models, except for the logistic model, which gave an exceptional low *y*_0_ value of 0.000 (fixed at bound) for growth curve starting at 10^6^ CFU mL^−1^. Logistic model gave values of SSE higher than 0.1 for all three growth curves of* E. coli*, which were almost twice the values obtained for any other growth curve shown in Table 2, 3, or 4. In this case all *r* ^2^ values were higher than 0.99 for all growth curves and all models, including the three-phase linear model.

After the results shown in Figure 1 and Tables 2, 3, and 4, the growth models selected for the rest of the investigation were three-phase linear, Gompertz, and Baranyi. The three selected models provided expected values for the growth parameters and fitted data properly, as shown by the statistics analysed, even in the case of the three-phase linear model, which was the one to provide worse goodness of fit, that is, lowest *r* ^2^ and highest RMSE values. Richards and logistic models were disregarded because they were not able to fit properly all these typical growth curves; that is, they gave abnormal values for growth parameters in some occasions.

Each model had a trend in providing higher or lower values, three-phase linear model giving consistently the lowest values, followed by Baranyi and Gompertz models, in this order, for both the growth rate and the lag phase. In a previous comparison of these three models, Buchanan et al. [2] already noticed and explained this effect on the basis of the nature of each model. These authors also highlighted the correlation existing between lag phase duration and specific growth rate values. This gives an explanation for the differences found in the values provided for growth rate and lag phase among these predictive models.

Hence, it seems that, depending on the predictive model chosen, values for growth rates and lag times will be consistently higher or lower. Then the question is which growth model can be considered the best for describing the true population growth and why. This can be answered with a deep analysis of the analytical aspects of the models, mechanistic elements, number of parameters, fitting properties, and so forth. However, such analyses have already been performed in the past [3], but researchers continue to use and compare different growth models [5, 12], probably because concluding results have not been reached yet.

In this context, our viewpoint is that the best performing model is the one which, for the growth parameters of different cultures of one microbial strain growing under exactly the same conditions and with the same precultural history, gives closer values.

With this purpose, an extensive analysis of growth curves was performed with the three selected growth models. Only growth rate was considered at this stage, since this parameter should be similar for all growth curves of the same microbial strain, even when starting from different initial inocula levels. A total of 345 individual growth curves were analysed, including those individual growth curves used to build the average OD growth curves shown in Figure 1.

Table 5 shows, as an example, growth rate values obtained for all* E. coli *individual growth curves with three-phase linear, Gompertz, and Baranyi growth models. With these data and those for the individual growth curves of* B. cereus* and* L. monocytogenes,* extensive statistical analyses were performed, including analyses of variance, medians, and quartiles. Analyses of variance showed that initial concentration did not influence growth rate and that significant differences were found among growth rate values given by the different models, as already pointed out. The statistical analyses also showed that, within models, growth rate data were not normally distributed, and significant differences were also found among median growth rate values.

**Figure 2:**
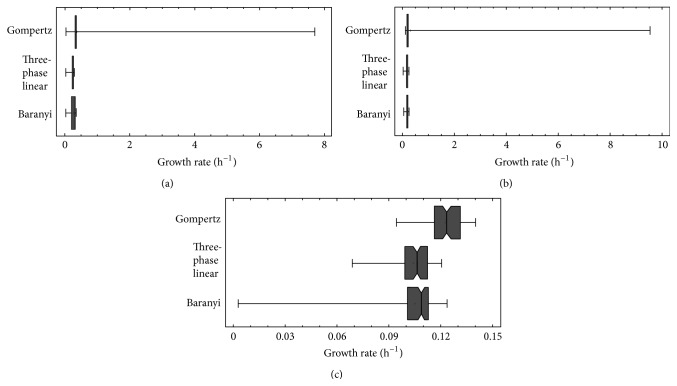
Box and whiskers plots for growth rate values of* Bacillus cereus *INRA-AVTZ 415 at 30°C in BHI (a),* Listeria monocytogenes *CECT 4031 at 37°C in TSB+YE (b), and* Escherichia coli *CECT 515 at 37°C in pH 5 TSB+YE (c).

The last step of this research was to analyse variation of growth rate values.  Table 6 shows average and standard deviation for growth rate of all three microorganisms as obtained by three-phase linear, Gompertz, and Baranyi models, and Figure 2 shows box and whiskers plots for the growth rate of the three microorganisms. These plots are based on the median and withstand perturbations caused by outliers better than plots based on the average. Since growth rate data were not normally distributed, box and whiskers plots are more appropriate than average and standard deviation shown in Table 6.

The results shown in Figure 2 clearly show that Gompertz model has a higher degree of variation in the growth rate values than the three-phase linear and Baranyi models. Gompertz model also generated some outlier growth rate values which extended the “whiskers” several units in the case of* B. cereus *and* L. monocytogenes. *It is certainly possible to obtain correct values for these outliers by changing the initial values when performing the nonlinear regression, but the purpose of this research was not to optimize the models, but to test them in order to choose the model that performs best. Hence initial values were not changed. Three-phase linear model provided slightly less variation than Baranyi, although the goodness of fit of this model is considerably worse than that of Baranyi model.

### 3.2. Plate Count Growth Curves

**Figure 3:**
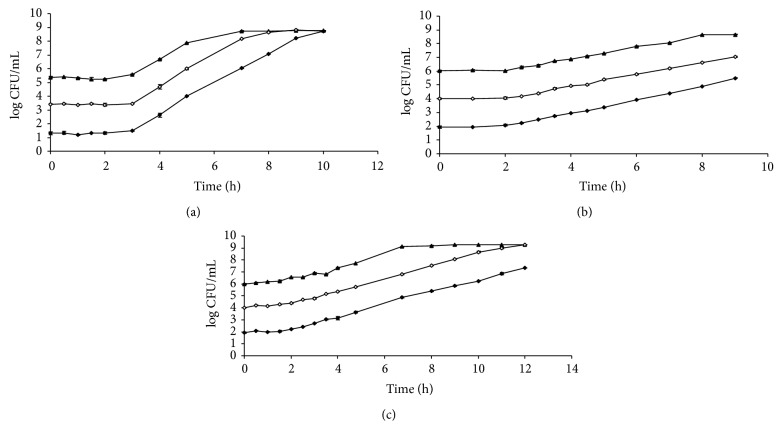
Plate count growth curves plotted with the average plate count values (±standard deviation) at each sampling time of* Bacillus cereus *INRA-AVTZ 415 at 30°C in BHI (a),* Listeria monocytogenes *CECT 4031 at 37°C in TSB+YE (b), and* Escherichia coli *CECT 515 at 37°C in pH 5 TSB+YE (c). Initial number of microorganisms: (⧫) 10^2^ CFU mL^−1^; (◊) 10^4^ CFU mL^−1^; (▲) 10^6^ CFU mL^−1^.

In order to double check the results obtained, several plate count growth curves of these same microorganisms were modelled with the five growth models.  Figure 3 shows the growth curves plotted with the average log CFU mL^−1^ at each sampling time of* B. cereus *INRA-AVTZ 415 at 30°C in BHI (a),* L. monocytogenes *CECT 4031 at 37°C in TSB+YE (b), and* E. coli *CECT 515 at 37°C in pH 5 TSB+YE (c) starting at different initial concentration of microorganisms. As usual for plate count growth curves, these curves included less data per curve and did not show data points in all growth phases. In this way, models were forced into the common situation of scarce data points. Tables 7, 8, and 9 show the growth parameters given by three-phase linear, Gompertz, logistic, Richards, and Baranyi models for the growth curves of* B. cereus, L. monocytogenes, *and* E. coli *shown in Figures 3(a), 3(b), and 3(c), respectively.

Similar results to those obtained with absorbance data were obtained when modelling the data from plate count growth curves. Three-phase linear model gave the lowest values for growth rates and lag times, followed by Baranyi, Gompertz, and logistic, in that order, for all three microorganisms. For these plate count growth curves, Richards model was not able to provide satisfactory values for growth rate for any of the curves. It is worth noting that some of the growth curves did not reach the stationary growth phase, and both three-phase linear and Baranyi models did not provide values for maximum population levels in these cases, while all other models provided uncertain estimations for this parameter. Again, three-phase linear was the model which had the worst goodness of fit, with *r* ^2^ values as low as 0.92 and RMSE values as high as 0.49.

Table 10 shows average and standard deviation for growth rate of all three microorganisms as obtained by three-phase linear, Gompertz, logistic, and Baranyi models with plate count growth curves. Again, when comparing the similarities in the growth rate values (Table 10), three-phase linear was the model to give less variation (lower standard deviation) for the different growth curves.

Comparisons of the behaviour of different growth models reported in literature have reached different conclusions. Zwietering et al. [3] studied the growth of* Lactobacillus plantarum *in MRS medium at different temperatures and concluded that the Gompertz model was the best-fitting model. When these authors [3] extended the study to several microorganisms they reached similar conclusions. Buchanan et al. [2] reported that the three-phase linear model was more robust than the Gompertz and Baranyi models in terms of successfully fitting growth curve data. In their research [2] they fitted experimental data for* E. coli *O157:H7. Schepers et al. [27] found that the Richards model was the best growth model for* Lactobacillus helveticus *grown at different pH values, and Dalgaard and Koutsoumanis [7] also agreed that Richards model gave the best estimates for absorbance growth curves obtained with mixtures of different microbial strains isolated from spoiled seafood and incubated in different conditions to obtain a wide range of growth yields. López et al. [5] concluded, after a detailed statistical evaluation, that the Baranyi model showed the best behaviour for the growth curves studied. Baranyi and three-phase linear models showed the best fit for plate count data of* Yersinia enterocolitica *grown under different conditions of pH, temperature, and CO_2_. Richards model was the best-fitting optical density data of different bacterial and fungal species grown under different conditions. However, Mytilinaios et al. [12] found more recently that Baranyi was the most capable model to fit optical density data obtained for* L. monocytogenes *at different temperatures and pH values. The Weibull model also adequately described microbial growth [5]. Baty and Delignette-Muller [4] found that Baranyi was the best curve-fitting model for most curves, although they noted that the intermodel variability was frequently minor comparing to the imprecision of the parameter estimates, due to the low quantity and quality of the data used to build the growth curves. Actually, the low quality data used by these authors correspond to the datasets tabulated in the work by Buchanan et al. [2]. Pal et al. [28] also showed that Baranyi model provided the best fit for a majority of growth curves obtained for* L. monocytogenes *at low temperatures in liquid cultures, although there was no significant difference among all the primary growth models analysed. Maybe the use of so many microorganisms growing under such different growth conditions could explain, at least in part, the differences in the conclusions reached by authors regarding the best-fitting model.

## 4. Conclusions

Our results show that both Baranyi and three-phase linear models provide low variability for growth rate values when analysing similar growth curves, hence being the models of choice. Three-phase linear model gives the lowest variation for growth rates, while Baranyi gives a variation marginally higher, despite much better overall fitting.

These results provide insight into predictive microbiology and will help food microbiologists and researchers to choose the proper primary growth predictive model.

## Figures and Tables

**Table 1:** Primary growth models.

Model	Equation^a^
Three-phase linear	*y* = *y*_0_ *t* ≤ *λ*
	*y* = *y*_0_ + *μ*(*t* − *λ*) *λ* < *t* < *t*_*s*_
	*y* = *y*_max⁡_ *t* ≥ *t*_*s*_

Gompertz	*y* = *y*_0_ + *C*(*e* ^(−*e*^(μ*e*(λ−*t*)/*C*+1)^)^)

Logistic	$y=y_{0}+\frac{C}{1+e^{\left(4\mu (\lambda -t)/C+2\right)}}$

Richards	$y=y_{0}+\frac{C}{{\left(1+\beta e^{1+\beta }e^{\left((\mu /C)(1+\beta )(1+1/\beta )(\lambda -t)\right)}\right)}^{1/\beta }}$

Baranyi	$y(t)=y_{0}+\mu A(t)-\ln\left(1+\frac{e^{\mu A(t)}-1}{e^{C}}\right)$ $A(t)=t+\frac{1}{\mu }\ln\left(e^{-\mu t}+e^{-\mu \lambda }-e^{-\mu (t+\lambda )}\right)$

^a^ *y*: log count or absorbance at time *t*; *y*_0_: initial log count or absorbance; *μ*: maximum growth rate; *λ*: lag time; *t*_*s*_: time to reach stationary growth phase; *y*_max⁡_: final log count or absorbance; *C*: increase in log count or absorbance from *y*_0_ to *y*_max⁡_; *β*: model coefficient.

**Table 2:** Growth parameters and their 95% confidence limits and coefficients of determination (*r* ^2^), SSE, and RMSE of fit obtained with different growth models for the average OD growth curves of *Bacillus cereus* INRA-AVTZ 415 at 30°C in BHI inoculating 10^2^, 10^4^, and 10^6^ CFU mL^−1^ shown in Figure 1(a).

Growth model	Initial concentration (CFU mL^−1^)	Lag time (h)	Growth rate (OD units h^−1^)	*y*_0_ (OD units)	*C* (OD units)	*β*	*r* ^2^	SSE	RMSE
Three-phase linear	10^2^	7.81 (7.68–7.94)	0.269 (0.253–0.285)	0.130 (0.042–0.217)	1.056		0.994	0.0034	0.0207
	10^4^	5.47 (5.29–5.66)	0.304 (0.274–0.334)	0.140 (0.019–0.261)	0.999		0.989	0.0042	0.0268
	10^6^	2.82 (2.68–2.95)	0.293 (0.273–0.312)	0.180 (0.027–0.332)	0.975		0.994	0.0032	0.0215

Gompertz	10^2^	7.95 (7.87–8.03)	0.312 (0.301–0.329)	0.117 (0.109–0.124)	1.107 (1.095–1.120)		0.999	0.0166	0.0179
	10^4^	5.50 (5.35–5.64)	0.342 (0.314–0.370)	0.118 (0.103–0.133)	1.078 (1.050–1.106)		0.996	0.0281	0.0283
	10^6^	2.87 (2.69–3.04)	0.349 (0.315–0.382)	0.142 (0.119–0.166)	1.057 (1.019–1.095)		0.995	0.0252	0.0311

Logistic	10^2^	8.01 (7.97–8.06)	0.313 (0.307–0.319)	0.105 (0.102–0.109)	1.104 (1.099–1.110)		0.999	0.0031	0.0077
	10^4^	5.56 (5.47–5.65)	0.345 (0.329–0.360)	0.105 (0.096–0.150)	1.070 (1.056–1.085)		0.999	0.0081	0.0152
	10^6^	2.87 (2.75–2.99)	0.347 (0.329–0.365)	0.114 (0.098–0.110)	1.066 (1.045–1.088)		0.999	0.0070	0.0164

Richards	10^2^	8.00 (7.96–8.05)	0.303 (0.285–0.321)	0.106 (0.103–0.110)	1.104 (1.099–1.110)	0.911 (0.769–1.054)	0.999	0.0030	0.0076
	10^4^	5.63 (5.54–5.72)	0.401 (0.373–0.429)	0.096 (0.086–0.100)	1.071 (1.058–1.084)	1.693 (1.257–2.130)	0.999	0.0060	0.0132
	10^6^	2.85 (2.75–2.95)	0.420 (0.400–0.439)	0.071 (0.048–0.090)	1.099 (1.076–1.122)	2.490 (1.823–3.158)	0.999	0.0029	0.0108

Baranyi	10^2^	7.80 (7.70–7.90)	0.275 (0.264–0.286)	0.155	1.047 (1.040–1.054)		0.999		0.0155
	10^4^	5.40 (5.29–5.51)	0.307 (0.291–0.323)	0.141	1.020 (1.010–1.030)		0.999		0.0178
	10^6^	2.76 (2.66–2.87)	0.312 (0.298–0.326)	0.152	1.015 (1.006–1.023)		0.999		0.0148

**Table 3:** Growth parameters and their 95% confidence limits and coefficients of determination (*r* ^2^), SSE, and RMSE of fit obtained with different growth models for the average OD growth curves of *Listeria monocytogenes* CECT 4031 at 37°C in TSB+YE inoculating 10^2^, 10^4^, and 10^6^ CFU mL^−1^ shown in Figure 1(b).

Growth model	Initial concentration (CFU mL^−1^)	Lag time (h)	Growth rate (OD units h^−1^)	*y*_0_ (OD units)	*C* (OD units)	*β*	*r* ^2^	SSE	RMSE
Three-phase linear	10^2^	19.11 (18.96–19.27)	0.163 (0.154–0.173)	0.142 (0.069–0.214)	0.742		0.994	0.0013	0.0127
	10^4^	14.61 (14.42–14.81)	0.173 (0.159–0.187)	0.143 (0.065–0.222)	0.724		0.989	0.0026	0.0181
	10^6^	8.79 (8.23–9.35)	0.150 (0.126–0.174)	0.149 (0.072–0.227)	0.712		0.930	0.0490	0.0614

Gompertz	10^2^	18.99 (18.87–19.10)	0.169 (0.161–0.177)	0.132 (0.128–0.136)	0.784 (0.776–0.792)		0.998	0.0221	0.0153
	10^4^	14.46 (14.31–14.62)	0.179 (0.167–0.191)	0.133 (0.127–0.139)	0.757 (0.746–0.768)		0.997	0.0353	0.0204
	10^6^	9.33 (9.09–9.56)	0.218 (0.190–0.245)	0.141 (0.129–0.154)	0.755 (0.726–0.783)		0.990	0.0491	0.0320

Logistic	10^2^	19.06 (18.99–19.12)	0.168 (0.163–0.171)	0.128 (0.126–0.130)	0.779 (0.775–0.783)		0.999	0.0052	0.0074
	10^4^	14.55 (14.43–14.67)	0.178 (0.170–0.186)	0.128 (0.123–0.132)	0.755 (0.748–0.762)		0.999	0.0160	0.0137
	10^6^	9.40 (9.22–9.58)	0.217 (0.199–0.235)	0.135 (0.126–0.144)	0.747 (0.730–0.765)		0.996	0.0227	0.0218

Richards	10^2^	19.12 (19.05–19.19)	0.183 (0.175–0.191)	0.126 (0.124–0.128)	0.778 (0.774–0.781)	1.333 (1.152–1.514)	0.999	0.0045	0.0069
	10^4^	14.74 (14.61–14.87)	0.219 (0.205–0.233)	0.123 (0.119–0.128)	0.754 (0.748–0.760)	2.040 (1.565–2.515)	0.999	0.0123	0.0121
	10^6^	9.93 (9.81–10.06)	0.318 (0.303–0.333)	0.119 (0.115–0.123)	0.748 (0.742–0.754)	6.730 (4.925–8.536)	0.999	0.0028	0.0078

Baranyi	10^2^	18.79 (18.65–18.93)	0.148 (0.142–0.155)	0.120	0.781 (0.776–0.786)		0.998		0.0138
	10^4^	14.04 (13.87–14.21)	0.163 (0.153–0.173)	0.130	0.745 (0.739–0.752)		0.997		0.0178
	10^6^	8.95 (8.75–9.15)	0.201 (0.183–0.218)	0.139	0.731 (0.718–0.743)		0.995		0.0219

**Table 4:** Growth parameters obtained and their 95% confidence limits and coefficients of determination (*r* ^2^), SSE, and RMSE of fit with different growth models for the average OD growth curves of *Escherichia coli* CECT 515 at 37°C in pH 5 TSB+YE inoculating 10^2^, 10^4^, and 10^6^ CFU mL^−1^ shown in Figure 1(c).

Growth model	Initial concentration (CFU mL^−1^)	Lag time (h)	Growth rate (OD units h^−1^)	*y*_0_ (OD units)	*C* (OD units)	*β*	*r* ^2^	SSE	RMSE
Three-phase linear	10^2^	14.80 (14.60–15.00)	0.103 (0.100–0.105)	0.161 (0.115–0.207)	1.622		0.995	0.0200	0.0250
	10^4^	9.70 (9.53–9.86)	0.110 (0.107–0.112)	0.167 (0.111–0.223)	1.656		0.996	0.0150	0.0220
	10^6^	4.44 (4.27–4.61)	0.111 (0.108–0.113)	0.176 (0.113–0.239)	1.600		0.996	0.0150	0.0224

Gompertz	10^2^	15.46 (15.29–15.62)	0.117 (0.115–0.119)	0.144 (0.137–0.152)	1.794 (1.780–1.807)		0.999	0.0700	0.0227
	10^4^	10.29 (10.13–10.46)	0.125 (0.123–0.128)	0.146 (0.137–0.155)	1.799 (1.785–1.812)		0.999	0.0663	0.0227
	10^6^	4.65 (4.42–4.89)	0.125 (0.122–0.127)	0.123 (0.106–0.140)	1.783 (1.761–1.805)		0.999	0.0597	0.0237

Logistic	10^2^	15.51 (15.12–15.90)	0.117 (0.113–0.121)	0.105 (0.088–0.121)	1.784 (1.758–1.810)		0.997	0.2544	0.0433
	10^4^	9.95 (9.56–10.33)	0.123 (0.119–0.126)	0.078 (0.057–0.094)	1.834 (1.808–1.860)		0.997	0.1825	0.0376
	10^6^	3.67 (3.46–3.89)	0.120 (0.117–0.123)	0.000^a^	1.875 (1.864–1.887)		0.997	0.1232	0.0339

Richards	10^2^	15.46 (15.18–15.73)	0.00008 (−0.016–0.016)	0.144 (0.137–0.152)	1.794 (1.772–1.816)	0.00024 (−0.049–0.049)	0.999	0.0670	0.0228
	10^4^	10.29 (9.98–10.61)	0.0001 (−0.063–0.063)	0.146 (0.135–0.156)	1.799 (1.778–1.819)	0.00041 (−0.185–0.186)	0.999	0.0663	0.0228
	10^6^	4.65 (4.41–4.90)	0.0002 (−0.107–0.107)	0.123 (0.086–0.159)	1.783 (1.743–1.824)	0.00062 (−0.316–0.317)	0.999	0.0598	0.0239

Baranyi	10^2^	14.31 (13.87–14.75)	0.101 (0.097–0.105)	0.126	1.742 (1.727–1.756)		0.996		0.0445
	10^4^	8.96 (8.50–9.41)	0.107 (0.103–0.111)	0.115	1.777 (1.763–1.789)		0.996		0.0445
	10^6^	2.72 (2.07–3.38)	0.104 (0.099–0.109)	0.060	1.791 (1.776–1.806)		0.994		0.0483

^a^Value fixed at bound.

**Table 5:** Growth rate values and their 95% confidence limits and coefficients of determination (*r* ^2^) and RMSE of fit obtained with three-phase linear, Gompertz, and Baranyi growth models for the growth curves of *Escherichia coli* CECT 515 at 37°C in pH 5 TSB+YE inoculating 10^2^, 10^4^, and 10^6^ CFU mL^−1^.

Initial concentration (CFU mL^−1^)	Three-phase linear model			Gompertz model			Baranyi model		
	Growth rate	*r* ^2^	RMSE	Growth rate	*r* ^2^	RMSE	Growth rate	*r* ^2^	RMSE
	(OD units h^−1^)			(OD units h^−1^)			(OD units h^−1^)		
10^2^	0.076 (0.073–0.078)	0.988	0.0425	0.094 (0.092–0.097)	0.997	0.0346	0.091	0.993	0.0516
	0.086 (0.084–0.088)	0.994	0.0295	0.100 (0.097–0.102)	0.998	0.0296	0.112	0.995	0.0469
	0.091 (0.089–0.093)	0.995	0.0284	0.106 (0.103–0.108)	0.998	0.0283	0.112	0.995	0.0478
	0.100 (0.096–0.105)	0.982	0.0516	0.118 (0.115–0.121)	0.998	0.0317	0.112	0.995	0.0456
	0.082 (0.078–0.085)	0.990	0.0414	0.100 (0.097–0.102)	0.998	0.0319	0.116	0.996	0.0412
	0.091 (0.088–0.094)	0.988	0.0478	0.114 (0.112–0.116)	0.999	0.0203	0.115	0.995	0.0453
	0.095 (0.093–0.097)	0.994	0.0348	0.113 (0.112–0.115)	0.999	0.0179	0.113	0.995	0.0482
	0.097 (0.095–0.099)	0.996	0.0230	0.111 (0.109–0.113)	0.999	0.0239	0.113	0.995	0.0465
	0.102 (0.100–0.104)	0.998	0.0173	0.116 (0.114–0.118)	0.999	0.0204	0.111	0.995	0.0489
	0.087 (0.085–0.089)	0.994	0.0300	0.100 (0.098–0.103)	0.998	0.0297	0.114	0.995	0.0475
	0.109 (0.107–0.111)	0.997	0.0215	0.125 (0.122–0.127)	0.999	0.0217	0.109	0.995	0.0458
	0.111 (0.108–0.114)	0.995	0.0285	0.126 (0.124–0.129)	0.999	0.0234	0.111	0.995	0.0444
	0.114 (0.111–0.117)	0.994	0.0319	0.132 (0.129–0.136)	0.999	0.0269	0.113	0.994	0.0499
	0.110 (0.106–0.114)	0.986	0.0516	0.132 (0.129–0.136)	0.999	0.0286	0.109	0.993	0.0543
	0.116 (0.113–0.119)	0.994	0.0330	0.136 (0.133–0.139)	0.999	0.0224	0.104	0.994	0.0516
	0.104 (0.101–0.106)	0.995	0.0318	0.120 (0.117–0.123)	0.998	0.0292	0.003	0.156	0.9944
	0.107 (0.105–0.109)	0.997	0.0223	0.122 (0.120–0.124)	0.999	0.0215	0.110	0.995	0.0465
	0.106 (0.104–0.109)	0.994	0.0332	0.122 (0.120–0.125)	0.999	0.0297	0.115	0.996	0.0443
	0.106 (0.103–0.108)	0.993	0.0378	0.123 (0.120–0.126)	0.999	0.0264	0.109	0.995	0.0456
	0.112 (0.106–0.118)	0.977	0.0629	0.136 (0.132–0.139)	0.999	0.0282	0.091	0.993	0.0501
	0.085 (0.083–0.087)	0.990	0.0415	0.106 (0.104–0.108)	0.999	0.0240	0.083	0.991	0.0576
	0.108 (0.106–0.110)	0.997	0.0223	0.121 (0.119–0.123)	0.999	0.0217	0.103	0.994	0.0499
	0.084 (0.081–0.088)	0.984	0.0507	0.098 (0.095–0.102)	0.996	0.0440	0.098	0.994	0.0500
	0.108 (0.106–0.110)	0.996	0.0260	0.125 (0.122–0.128)	0.999	0.0261	0.109	0.995	0.0458
	0.108 (0.106–0.111)	0.996	0.0277	0.125 (0.123–0.128)	0.999	0.0262	0.098	0.993	0.0525
	0.110 (0.106–0.114)	0.988	0.0484	0.132 (0.129–0.135)	0.999	0.0257	0.101	0.994	0.0498
	0.109 (0.106–0.111)	0.994	0.0338	0.128 (0.125–0.131)	0.999	0.0259	0.102	0.993	0.0517
	0.102 (0.100–0.104)	0.995	0.0264	0.116 (0.114–0.119)	0.999	0.0252	0.102	0.993	0.0536
	0.104 (0.101–0.108)	0.991	0.0394	0.121 (0.118–0.124)	0.998	0.0329	0.104	0.994	0.0499
	0.118 (0.115–0.121)	0.996	0.0275	0.137 (0.135–0.140)	0.999	0.0216	0.095	0.993	0.0510

10^4^	0.090 (0.088–0.093)	0.993	0.0331	0.105 (0.102–0.107)	0.998	0.0319	0.090	0.996	0.0432
	0.099 (0.096–0.102)	0.991	0.0429	0.120 (0.117–0.1229)	0.998	0.0292	0.106	0.996	0.0437
	0.102 (0.099–0.104)	0.995	0.0283	0.117 (0.115–0.119)	0.999	0.0283	0.101	0.996	0.0477
	0.101 (0.099–0.103)	0.995	0.0304	0.117 (0.115–0.120)	0.998	0.0290	0.104	0.996	0.0418
	0.101 (0.099–0.104)	0.995	0.0283	0.115 (0.112–0.118)	0.998	0.0304	0.102	0.996	0.0452
	0.102 (0.100–0.104)	0.996	0.0295	0.118 (0.115–0.121)	0.998	0.0302	0.105	0.996	0.0433
	0.095 (0.093–0.098)	0.989	0.0464	0.116 (0.113–0.119)	0.998	0.0308	0.101	0.996	0.0476
	0.101 (0.098–0.103)	0.995	0.0279	0.115 (0.112–0.117)	0.998	0.0283	0.100	0.996	0.0450
	0.102 (0.100–0.104)	0.996	0.0257	0.116 (0.114–0.118)	0.999	0.0216	0.100	0.995	0.0477
	0.074 (0.070–0.079)	0.950	0.1039	0.116 (0.113–0.118)	0.999	0.0249	0.100	0.995	0.0479
	0.094 (0.091–0.096)	0.993	0.0315	0.110 (0.108–0.113)	0.998	0.0264	0.096	0.995	0.0465
	0.098 (0.095–0.100)	0.992	0.0431	0.118 (0.115–0.120)	0.999	0.0258	0.103	0.996	0.0431
	0.113 (0.111–0.116)	0.996	0.0264	0.131 (0.128–0.134)	0.999	0.0267	0.116	0.997	0.0427
	0.111 (0.109–0.114)	0.995	0.0278	0.126 (0.123–0.130)	0.998	0.0286	0.111	0.996	0.0453
	0.108 (0.106–0.110)	0.997	0.0231	0.123 (0.121–0.126)	0.999	0.0244	0.108	0.996	0.0444
	0.112 (0.109–0.115)	0.992	0.0362	0.128 (0.124–0.131)	0.998	0.0338	0.113	0.996	0.0452
	0.110 (0.107–0.112)	0.996	0.0256	0.125 (0.122–0.128)	0.999	0.0280	0.111	0.996	0.0429
	0.105 (0.102–0.107)	0.995	0.0302	0.123 (0.121–0.126)	0.999	0.0239	0.107	0.996	0.0460
	0.111 (0.107–0.116)	0.982	0.0638	0.140 (0.137–0.143)	0.999	0.0275	0.124	0.996	0.0443
	0.119 (0.117–0.121)	0.997	0.0225	0.137 (0.134–0.140)	0.999	0.0265	0.122	0.997	0.0400
	0.117 (0.113–0.120)	0.992	0.0399	0.140 (0.137–0.143)	0.999	0.0236	0.123	0.996	0.0454
	0.120 (0.118–0.122)	0.998	0.0218	0.138 (0.135–0.142)	0.999	0.0260	0.123	0.997	0.0401
	0.120 (0.117–0.122)	0.996	0.0274	0.137 (0.134–0.140)	0.999	0.0271	0.120	0.996	0.0471
	0.116 (0.113–0.119)	0.995	0.0304	0.132 (0.129–0.135)	0.999	0.0249	0.117	0.997	0.0397
	0.120 (0.117–0.123)	0.994	0.0329	0.137 (0.133–0.141)	0.998	0.0326	0.123	0.997	0.0424
	0.117 (0.114–0.119)	0.997	0.0247	0.135 (0.132–0.138)	0.999	0.0239	0.121	0.997	0.0374
	0.105 (0.101–0.109)	0.980	0.0715	0.137 (0.133–0.141)	0.998	0.0292	0.123	0.997	0.0371
	0.112 (0.110–0.114)	0.997	0.0216	0.128 (0.125–0.130)	0.999	0.0218	0.112	0.996	0.0458
	0.120 (0.117–0.122)	0.996	0.0278	0.137 (0.134–0.140)	0.999	0.0249	0.120	0.996	0.0449
	0.109 (0.106–0.112)	0.993	0.0318	0.124 (0.121–0.126)	0.999	0.0246	0.105	0.994	0.0531

10^6^	0.092 (0.089–0.096)	0.989	0.0400	0.112 (0.109–0.114)	0.998	0.0269	0.091	0.993	0.0516
	0.114 (0.111–0.117)	0.994	0.0302	0.130 (0.126–0.134)	0.998	0.0325	0.112	0.995	0.0469
	0.101 (0.096–0.105)	0.979	0.0667	0.130 (0.127–0.134)	0.998	0.0278	0.112	0.995	0.0478
	0.115 (0.112–0.117)	0.996	0.0248	0.129 (0.126–0.132)	0.998	0.0267	0.112	0.995	0.0456
	0.112 (0.109–0.115)	0.995	0.0310	0.131 (0.128–0.135)	0.998	0.0303	0.116	0.996	0.0412
	0.115 (0.113–0.117)	0.996	0.0245	0.132 (0.129–0.135)	0.999	0.0259	0.115	0.995	0.0453
	0.117 (0.114–0.119)	0.997	0.0243	0.131 (0.128–0.135)	0.998	0.0280	0.113	0.995	0.0482
	0.113 (0.110–0.115)	0.995	0.0319	0.130 (0.126–0.134)	0.998	0.0321	0.113	0.995	0.0465
	0.114 (0.112–0.117)	0.995	0.0294	0.130 (0.126–0.133)	0.998	0.0298	0.111	0.995	0.0489
	0.114 (0.111–0.117)	0.995	0.0282	0.132 (0.129–0.134)	0.998	0.0284	0.114	0.995	0.0475
	0.111 (0.108–0.113)	0.997	0.0223	0.127 (0.124–0.130)	0.998	0.0269	0.109	0.995	0.0458
	0.112 (0.109–0.115)	0.995	0.0292	0.128 (0.125–0.132)	0.998	0.0309	0.111	0.995	0.0444
	0.116 (0.114–0.118)	0.997	0.0227	0.131 (0.128–0.135)	0.998	0.0261	0.113	0.994	0.0499
	0.069 (0.063–0.074)	0.903	0.1575	0.129 (0.127–0.132)	0.999	0.0248	0.109	0.993	0.0543
	0.108 (0.106–0.111)	0.996	0.0250	0.123 (0.120–0.126)	0.999	0.0243	0.104	0.994	0.0516
	0.120 (0.117–0.122)	0.995	0.0289	0.137 (0.133–0.140)	0.998	0.0291	0.003	0.156	0.9944
	0.112 (0.110–0.115)	0.996	0.0250	0.127 (0.124–0.131)	0.998	0.0289	0.110	0.995	0.0465
	0.113 (0.111–0.116)	0.996	0.0279	0.132 (0.129–0.136)	0.998	0.0284	0.115	0.996	0.0443
	0.102 (0.098–0.106)	0.987	0.0494	0.126 (0.123–0.129)	0.998	0.0261	0.109	0.995	0.0456
	0.086 (0.082–0.089)	0.982	0.0543	0.109 (0.106–0.112)	0.998	0.0278	0.091	0.993	0.0501
	0.079 (0.076–0.083)	0.975	0.0603	0.101 (0.098–0.105)	0.997	0.0353	0.083	0.991	0.0576
	0.106 (0.104–0.109)	0.995	0.0286	0.122 (0.119–0.125)	0.998	0.0281	0.103	0.994	0.0499
	0.102 (0.099–0.105)	0.993	0.0306	0.117 (0.114–0.119)	0.998	0.0261	0.098	0.994	0.0500
	0.111 (0.108–0.113)	0.996	0.0234	0.127 (0.124–0.130)	0.999	0.0251	0.109	0.995	0.0458
	0.102 (0.099–0.105)	0.992	0.0345	0.119 (0.116–0.121)	0.999	0.0240	0.098	0.993	0.0525
	0.104 (0.101–0.107)	0.993	0.0293	0.119 (0.116–0.122)	0.998	0.0258	0.101	0.994	0.0498
	0.071 (0.066–0.076)	0.931	0.1209	0.121 (0.118–0.124)	0.998	0.0250	0.102	0.993	0.0517
	0.105 (0.102–0.108)	0.994	0.0312	0.122 (0.119–0.125)	0.998	0.0266	0.102	0.993	0.0536
	0.106 (0.103–0.109)	0.993	0.0341	0.123 (0.120–0.126)	0.998	0.0270	0.104	0.994	0.0499
	0.094 (0.091–0.098)	0.989	0.0399	0.114 (0.111–0.117)	0.998	0.0292	0.095	0.993	0.0510

**Table 6:** Average ± standard deviation of growth rate values (OD units h^−1^) obtained with three-phase linear, Gompertz, and Baranyi growth models for all the growth curves of *Bacillus cereus* INRA-AVTZ 415 at 30°C in BHI, *Listeria monocytogenes* CECT 4031 at 37°C in TSB+YE, and *Escherichia coli* CECT 515 at 37°C in pH 5 TSB+YE.

Growth model	*B*. *cereus *	*L*. *monocytogenes *	*E*. *coli *
Three-phase linear	0.226 ± 0.062	0.170 ± 0.022	0.104 ± 0.011
Gompertz	0.364 ± 0.627	0.296 ± 0.941	0.123 ± 0.011
Baranyi	0.261 ± 0.085	0.164 ± 0.042	0.105 ± 0.018

**Table 7:** Growth parameters and their 95% confidence limits and coefficients of determination (*r* ^2^), SSE, and RMSE of fit obtained with different growth models for average plate count growth curves of *Bacillus cereus* INRA-AVTZ 415 at 30°C in BHI inoculating 10, 10^3^, and 10^5^ CFU mL^−1^ shown in Figure 3(a).

Growth model	Initial concentration (CFU mL^−1^)	Lag time (h)	Growth rate (log cycles h^−1^)	*y*_0_ (log CFU mL^−1^)	*C* (log cycles)	*β*	*r* ^2^	SSE	RMSE
Three-phase linear	10	2.75 (2.59–2.91)	1.112 (1.073–1.115)	1.343 (1.202–1.484)	7.701		0.993	0.2880	0.2400
	10^3^	2.92 (2.79–3.05)	1.182 (1.121–1.138)	3.414 (3.338–3.490)	5.337		0.927	0.7295	0.4931
	10^5^	2.77 (2.64–2.91)	1.143 (1.049–1.232)	5.367 (5.289–5.444)	3.372		0.920	0.8099	0.4025

Gompertz	10	3.06 (2.59–3.52)	1.276 (1.112–1.440)	1.211 (0.983–1.440)	9.049 (7.519–10.580)		0.996	0.2846	0.1886
	10^3^	3.30 (3.08–3.53)	1.598 (1.380–1.816)	3.403 (3.302–3.504)	5.521 (5.284–5.757)		0.998	0.0811	0.1007
	10^5^	3.00 (2.82–3.17)	1.386 (1.206–1.565)	5.319 (5.257–5.382)	3.469 (3.363–3.574)		0.999	0.0301	0.0613

Logistic	10	3.04 (2.12–3.96)	1.318 (1.081–1.556)	0.914 (0.408–1.421)	8.529 (6.904–10.150)		0.992	0.5385	0.2595
	10^3^	3.39 (3.00–3.79)	1.651 (1.296–2.006)	3.330 (3.154–3.507)	5.433 (5.112–5.754)		0.996	0.1747	0.1478
	10^5^	3.10 (2.84–3.36)	1.451 (1.193–1.709)	5.288 (5.194–5.381)	3.461 (3.320–3.603)		0.997	0.0551	0.0830

Richards	10	3.06 (1.92–4.19)	0.0006 (−4.543–4.544)	1.211 (0.779–1.643)	9.049 (5.176–12.920)	0.00017 (−1.310–1.311)	0.995	0.2846	0.2016
	10^3^	3.30 (3.05–3.55)	0.004 (−2.950–2.958)	3.403 (3.288–3.519)	5.520 (5.229–5.812)	0.0009 (−0.681–0.683)	0.998	0.0811	0.1070
	10^5^	3.02 (2.82–3.21)	0.536 (−1.052–2.125)	5.316 (5.247–5.385)	3.460 (3.350–3.571)	0.181 (−0.508–0.871)	0.998	0.0275	0.0627

Baranyi	10	2.88 (2.39–3.37)	1.187 (1.054–1.324)	1.172	7.869		0.996		0.1792
	10^3^	3.09 (2.77–3.41)	1.313 (1.137–1.324)	3.360	5.387		0.997		0.1248
	10^5^	2.95 (2.75–3.15)	1.275 (1.121–1.429)	5.309	3.436		0.999		0.0604

**Table 8:** Growth parameters and their 95% confidence limits and coefficients of determination (*r* ^2^), SSE, and RMSE of fit obtained with different growth models for average plate count growth curves of *Listeria monocytogenes* CECT 4031 at 37°C in TSB+YE inoculating 10^2^, 10^4^, and 10^6^ CFU mL^−1^ shown in Figure 3(b).

Growth model	Initial concentration (CFU mL^−1^)	Lag time (h)	Growth rate (log cycles h^−1^)	*y*_0_ (log CFU mL^−1^)	*C* (log cycles)	*β*	*r* ^2^	SSE	RMSE
Three-phase linear	10^2^	2.00 (1.85–2.14)	0.487 (0.467–0.506)	1.988 (1.820–2.150)	—		0.997	0.0346	0.0620
	10^4^	1.95 (1.75–2.06)	0.432 (0.424–0.441)	4.006 (3.970–4.040)	—		0.986	0.1525	0.1235
	10^6^	2.00 (1.78–2.24)	0.423 (0.402–0.441)	6.113 (5.870–6.360)	2.554		0.985	0.0916	0.1070

Gompertz	10^2^	2.13 (1.63–2.63)	0.542 (0.510–0.574)	1.803 (1.599–2.007)	6.071 (4.105–8.038)		0.997	0.0406	0.0672
	10^4^	2.22 (1.63–2.81)	0.503 (0.443–0.564)	3.932 (3.755–4.109)	3.917 (2.982–4.852)		0.993	0.0691	0.0870
	10^6^	2.27 (1.70–2.83)	0.485 (0.417–0.554)	6.009 (5.856–6.163)	3.227 (2.604–3.85)		0.992	0.0655	0.0853

Logistic	10^2^	1.73 (0.75–2.71)	0.559 (0.511–0.606)	1.501 (1.086–1.917)	5.297 (3.521–7.073)		0.995	0.0614	0.0826
	10^4^	1.91 (0.72–3.10)	0.513 (0.428–0.597)	3.715 (3.340–4.091)	3.739 (2.689–4.79)		0.989	0.1087	0.1099
	10^6^	2.12 (1.18–3.06)	0.501 (0.415–0.587)	5.863 (5.599–6.127)	3.073 (2.465–3.681)		0.990	0.0841	0.0967

Richards	10^2^	2.13 (1.41–2.85)	0.003 (−2.733–2.738)	1.803 (1.282–2.323)	6.068 (0.701–11.440)	0.002 (−1.865–1.868)	0.996	0.0407	0.0713
	10^4^	2.22 (1.57–2.86)	0.0008 (−2.303–2.304)	3.932 (3.614–4.250)	3.917 (2.384–5.450)	0.0006 (−1.686–1.687)	0.992	0.0691	0.0929
	10^6^	2.27 (1.70–2.84)	0.013 (0.011–0.015)	6.008 (5.854–6.163)	3.222 (2.601–3.843)	0.01^a^	0.992	0.0657	0.0854

Baranyi	10^2^	2.07 (1.84–2.31)	0.514 (0.494–0.534)	1.891	—		0.999		0.0453
	10^4^	1.86 (1.34–2.37)	0.447 (0.410–0.483)	3.896	—		0.993		0.0875
	10^6^	2.14 (1.64–2.64)	0.460 (0.402–0.517)	5.996	2.774		0.993		0.0774

^a^Value fixed at bound.

**Table 9:** Growth parameters and their 95% confidence limits and coefficients of determination (*r* ^2^), SSE, and RMSE of fit obtained with different growth models for average plate count growth curves of *Escherichia coli* CECT 4031 at 37°C in pH 5 TSB+YE inoculating 10^2^, 10^4^, and 10^6^ CFU mL^−1^ shown in Figure 3(c).

Growth model	Initial concentration (CFU mL^−1^)	Lag time (h)	Growth rate (log cycles h^−1^)	*y*_0_ (log CFU mL^−1^)	*C* (log cycles)	*β*	*r* ^2^	SSE	RMSE
Three-phase linear	10^2^	1.61 (1.41–1.81)	0.518 (0.507–0.526)	2.011 (1.800–2.220)	—		0.992	0.3897	0.1731
	10^4^	1.63 (1.39–1.89)	0.521 (0.508–0.544)	4.108 (3.790–4.430)	5.175		0.991	0.4079	0.1771
	10^6^	1.74 (1.45–2.02)	0.574 (0.523–0.627)	6.052 (5.920–6.180)	3.229		0.948	0.9450	0.2931

Gompertz	10^2^	1.49 (0.57–2.42)	0.576 (0.531–0.620)	1.687 (1.330–2.043)	7.489 (5.845–9.132)		0.997	0.1518	0.1125
	10^4^	1.55 (0.97–2.13)	0.580 (0.549–0.611)	3.862 (3.641–4.083)	7.252 (6.270–8.234)		0.999	0.0669	0.0747
	10^6^	2.25 (1.63–2.88)	0.692 (0.515–0.869)	6.180 (5.988–6.372)	3.233 (2.898–3.568)		0.985	0.3275	0.1652

Logistic	10^2^	0.41 (−1.52–2.34)	0.578 (0.520–0.635)	1.011 (0.223–1.799)	7.501 (5.528–9.474)		0.995	0.2043	0.1305
	10^4^	0.85 (−0.11–1.81)	0.593 (0.557–0.630)	3.344 (2.965–3.722)	6.952 (6.051–7.852)		0.998	0.0727	0.0778
	10^6^	2.28 (1.64–2.93)	0.728 (0.576–0.880)	6.065 (5.866–6.265)	3.264 (2.987–3.541)		0.992	0.1763	0.1212

Richards	10^2^	1.49 (−0.21–3.19)	0.002 (−3.220–3.224)	1.686 (0.453–2.919)	7.488 (4.798–10.180)	0.001 (−2.065–2.067)	0.996	0.1519	0.1175
	10^4^	1.53 (0.45–2.60)	0.061 (−1.745–1.867)	3.839 (3.100–4.578)	7.214 (5.605–8.822)	0.041 (−1.248–1.330)	0.998	0.0671	0.0781
	10^6^	2.11 (1.08–3.14)	0.953 (0.749–1.157)	5.573 (4.954–6.192)	3.691 (3.069–4.312)	7.363 (−3.876–18.600)	0.996	0.0874	0.0891

Baranyi	10^2^	1.32 (0.71–1.92)	0.523 (0.489–0.556)	1.807	—		0.996		0.1176
	10^4^	1.79 (1.45–1.92)	0.568 (0.539–0.598)	4.040	5.512		0.999		0.0624
	10^6^	2.35 (1.76–2.93)	0.687 (0.528–0.849)	6.181	3.093		0.991		0.1288

**Table 10:** Average ± standard deviation of growth rate values (log cycles h^−1^) obtained with three-phase linear, Gompertz, and Baranyi growth models for all the growth curves of *Bacillus cereus* INRA-AVTZ 415 at 30°C in BHI,* Listeria monocytogenes* CECT 4031 at 37°C in TSB+YE, and *Escherichia coli* CECT 515 at 37°C in pH 5 TSB+YE.

Growth model	*B*. *cereus *	*L*. *monocytogenes *	*E*. *coli *
Three-phase linear	1.146 ± 0.035	0.447 ± 0.035	0.538 ± 0.032
Gompertz	1.420 ± 0.164	0.510 ± 0.029	0.616 ± 0.066
Logistic	1.473 ± 0.168	0.524 ± 0.031	0.633 ± 0.083
Baranyi	1.258 ± 0.065	0.474 ± 0.036	0.593 ± 0.085
